# Downregulation of the Proinflammatory State of Circulating Mononuclear Cells by Short-Term Treatment with Pioglitazone in Patients with Type 2 Diabetes Mellitus and Coronary Artery Disease

**DOI:** 10.1155/2011/647017

**Published:** 2011-07-26

**Authors:** Andreas Pfützner, Alexander Weise, Elisabeth Pfützner-Riehn, Georg Lübben, Michael Morcos, Efstrathios Karagiannis, Matthias Weber, Thomas Forst

**Affiliations:** ^1^IKFE Institute for Clinical Research and Development, Research Laboratory, 55116 Mainz, Germany; ^2^University of Applied Sciences, Department of Natural Sciences, 53359 Rheinbach, Germany; ^3^The Department of Endocrinology and Metabolism, University Hospital of Mainz, 55131 Mainz, Germany; ^4^Takeda Pharma, Medical Department, 52066 Aachen, Germany; ^5^Cardiology Center, Medical Department, 69120 Heidelberg, Germany

## Abstract

*Background*. This study was performed to investigate the influence of a short-term treatment with pioglitazone versus placebo on inflammatory activation of mononuclear cells (mRNA expression/protein secretion of inflammatory markers). *Methods and Results*. Sixty-three patients with well-controlled type 2 diabetes (52 males, 11 females, age (Mean ± SD): 66 ± 7 yrs, disease duration: 6.6 ± 9.6 yrs, HbA1c: 6.7 ± 0.6%) were randomized to additional 45 mg of pioglitazone or placebo to their existing metformin and sulfonylurea therpay for four weeks in a double-blind study design. Protein risk marker levels (hsCRP, MMP-9, MCP-1, etc.) and the expression of NF*κ*B subunits and NF*κ*B-modulated cytokines from isolated peripheral monocyte/macrophages were determined at baseline and endpoint. There were no changes in HbA1c, but significant biomarker improvements were seen with pioglitazone only. The mRNA marker expression was downregulated by pioglitazone and further up-regulated with placebo (e.g., P105 pioglitazone: −19%/placebo: +6%, RelA: −20%/+2%, MMP−9: −36%/+9%, TNF*α*: −10%/+14%, *P* < 0.05 between groups in all cases). *Conclusions*. Pioglitazone very rapidly down-regulated the activated state of peripheral monocytes/macrophages as assessed by mRNA expression of NF*κ*B and NF*κ*B-modulated cytokines and decreased plasma levels of cardiovascular risk marker proteins independent of glycemic control.

## 1. Introduction

Obesity has been demonstrated to be associated with metabolic syndrome and cardiovascular disease, including severe complications, like acute coronary syndrome, myocardial infarction, and stroke [1, 2]. An increase in body weight is usually accompanied by an increase in oxidative stress [3] and an elevation in the tissue expression and plasma levels of proinflammatory cytokines, such as tumor necrosis factor-*α* (TNF*α*) [4], interleukin-6 (IL-6) [5, 6], plasminogen activator-inhibitor-1 (PAI-1) [7], and others [8]. This protein expression profile indicates the prevalence of a chronic systemic inflammation, and differentiating preadipocytes deriving from mesenchymal stem cells especially in the visceral lipid tissue are considered to be a major source for these cytokines and proteins [9]. It is believed that the crosstalk between the preadipocytes and other tissues contributes to a general up-regulation of the immune system, including an activation of circulating monocytes and macrophages, resulting in an increased risk for atherosclerosis and vascular disease [10, 11]. 

It has been demonstrated by Ghanim and Coworkers that circulating mononuclear cells in obese patients are in a proinflammatory state with an increase in intranuclear NF-*κ*B binding, a decrease in I*κ*B-ß, and an increase in the transcription of proinflammatory genes regulated by NF-*κ*B, including migration inhibitory factor (MIF), IL-6, TNF*α*, and matrix metalloproteinase 9 (MMP-9) [12]. The same group was able to demonstrate that an increased plasma concentration of MIF and an increased transcription of MIF mRNA in mononuclear cells, which was related to the body-mass index and hsCRP concentrations, could be reduced by a six-week treatment with metformin in eight nondiabetic patients with obesity. The authors concluded that metformin might have beneficial effects on cardiovascular mortality in patients with type 2 diabetes [13], which is in part confirmed by the few currently existing larger outcome trials on this topic [14, 15]. The same group also showed that the insulin-sensitizing drug troglitazone was able to suppress NF-*κ*B activity and stimulate I*κ*B in nondiabetic obese patients, which gave evidence for an anti-inflammatory effect of this drug [16]. Troglitazone was, however, taken from the market because of hepatotoxicity [17]. 

It has been shown in randomized prospective trials that treatment with pioglitazone, another agonists to the peroxisome proliferators-activated receptor *γ*, may improve clinical and laboratory surrogate markers for atherosclerosis and cardiovascular risk, like intima-media thickness, hsCRP, or MMP-9 independent of glycemic control [18–20], and that it may even improve macrovascular outcome in type 2 diabetic patients when used in secondary prevention [21–23]. The anti-inflammatory and antithrombotic effects of thiazolidinediones occur very rapidly and significantly earlier as compared to the metabolic and glycemic effects of these drugs [24, 25].

In this study, we explored the short-term effects of an addition of pioglitazone (versus placebo) to an existing effective oral antidiabetic therapy with metformin and/or sulfonylurea on the proinflammatory activation of circulating mononuclear cells in well-controlled patients with type 2 diabetes mellitus and elevated risk for atherosclerosis. For this purpose, we investigated the mRNA expression of the inhibitors to NF-*κ*B (I*κ*B-*α* and I*κ*B-*β*) [26], p105 (precursor to the p50 subunit), and Rel-A (p65 subunit) as measures for the quantity of intranuclear NF-*κ*B [27], and several proinflammatory mediators and markers that are known to be modulated by NF-*κ*B, such as TNF*α*, IL-6, MIF, and MMP-9 [5, 12, 28] before and after four weeks of treatment..

## 2. Patients and Methods

This investigation was performed as a double-blind, placebo-controlled, randomised multicentre study in patients with type 2 diabetes and established atherosclerosis. Inclusion criteria were an age between 20 and 80 years, an HbA1c < 8.5%, an angiographically confirmed coronary artery disease, and an activated chronic systemic inflammation (characterized by an increased hsCRP level ≥ 1 mg/L). Patients had to be on any stable oral antidiabetic treatment with the exception of a thiazolidinedione for at least 3 months. Main exclusion criteria were systemic inflammation of other origin, invasive cardiovascular intervention within the last 3 months, history of heart failure (NYHA I-IV), major hepatic or renal disease, and progressive fatal disease. The study was approved by the local ethical review board and the national regulatory agency, and all patients provided a written informed consent prior to study inclusion (registration number NCT00479986 at http://www.clinicaltrials.gov/). 

The patients were randomised by a telephone randomization procedure to either receive 45 mg pioglitazone or placebo in addition to their individual oral antidiabetic treatment for 4 weeks. Primary objective was the change from baseline of MMP9 levels as an indicator of macrophage activation. Secondary objectives were the changes of other indicators of chronic systemic inflammation (e.g., plasma hsCRP and MCP-1 levels, etc.) or insulin resistance as indicated in Table 2. Blood for the measurement of fasting glucose, MMP-9, and hsCRP was taken at baseline and after 3, 7, 10, 14, and 28 days of study treatment. Blood for assessment of the mRNA expression profile of circulating mononuclear cells as well as for assessment of circulating plasma levels of HbA1c, insulin, intact proinsulin, adiponectin, IL-6, sCD40L, P-selectin, MIF, Angiotensin II, complement factor 3, and blood lipids were obtained at baseline and at the end of the study. Insulin resistance was calculated using the HOMA_IR_ score at baseline and study endpoint as published previously [29, 30]. 

### 2.1. Laboratory Methods

HbA1c was measured by means of an HPLC method (Menarini, Neuss), and lipids were assessed by standard dry chemistry (Olympus, Hamburg, Germany). Immunoassays were applied to determine the plasma concentrations of insulin (CLIA, Invitreon, Cardiff, UK), intact proinsulin (CLIA, Invitreon, Cardiff, UK), adiponectin (RIA, Linco, St. Charles, MO), IL-6 (Elisa, IBL, Hamburg, Germany), MMP-9 (ELISA, R&D Systems, Wiesbaden, Germany), MCP-1 (ELISA, R&D Systems, Wiesbaden, Germany), sCD40L (ELISA, R&D Systems, Wiesbaden, Germany), P-selectin (R&D Systems, Wiesbaden, Germany), TNF*α* (ELISA, IBL, Hamburg, Germany), MIF (R&D Systems, Wiesbaden, Germany), Angiotensin II (ELISA, DRG-Diagnostics, Marburg, Germany), and complement factor C3 (ELISA, BioCat, Heidelberg, Germany).

### 2.2. Isolation of Mononuclear Cells and mRNA Extraction

Isolation of MNC from whole blood was performed as a density gradient centrifugation by means of the ACCUSPIN System-HISTOPAQUE-1077 (Sigma-Aldrich Chemie GmbH, Steinheim, Germany). The isolation of macrophages and monocytes from the collected cells was performed by MACS magnetic cell sorting with CD14 MicroBeads (human) (Miltenyi Biotec GmbH, Bergisch Gladbach, Germany). The CD14-positive (CD14+) cells (macrophages and monocytes) were first magnetically labelled with MACS CD14 MicroBeads. The cell suspension was loaded on an MS MACS Column which was placed in the magnetic field of a MiniMACS Separator. The magnetically labelled CD14+ cells were retained in the column and separated from the unlabelled cell fraction.

### 2.3. mRNA Isolation from Macrophages and Monocytes and Reverse Transcription

The mRNA isolation from macrophages and monocytes was performed with the High Pure RNA Isolation Kit (Roche Applied Science, Penzberg, Germany). The cells were first lysed and the intact and undegraded RNA was adsorbed to a glass fibre fleece. Simultaneously, RNAses were inactivated. Furthermore, residues of contaminating DNA were digested, and the RNA was purified from salts, proteins, and other impurities. Purity of the isolated mRNA was assessed by real-time PCR (LightCycler II, Roche Diagnostics, Mannheim, Germany). The transcription of mRNA in cDNA was performed on a thermocycler (Biozym Diagnostik GmbH, Oldendorf, Germany) according to a standardized protocol (“Transcriptor First Strand cDNA Synthesis Kit”, Instruction Manual Version 1, 2004, Roche Applied Science, Penzberg, Germany).

### 2.4. Primers for PCR Reactions

Sequence-specific primers were designed by TIB MOLBIOL (Syntheselabor GmbH, Berlin, Germany) to amplify the gene sequences of Rel-A, p105, I*κ*B-*α*, I*κ*B-ß, and IL-6. The primers for TNF-*α* were taken from “Rapid Cycle Real-Time PCR Methods and Applications Quantification” [31]. The primers for MIF and MMP9 were reproduced from previous reports [12, 32]. A list of the primers used for the quantification experiments, the primer-specific PCR protocols, and the specific amplification product melting points are provided in Table 1. An additional agarose gel electrophoresis assay was performed to verify the correct length of the amplification products.

### 2.5. Quantification of mRNA Expression Profiles

The RNA quantification in this study was performed by means of a calibrator-normalized relative quantification method based on the LightCycler II system (Roche Diagnostics, Mannheim, Germany), where quantification of a target and a reference gene is a function of PCR efficiency and the sample crossing point. The sample crossing point is the amplification cycle during an amplification assay, at which the fluorescence of a probe rises above background fluorescence. This occurs usually at the second derivative maximum (fastest change in fluorescence). The calibrator, a positive sample for the investigated gene product, must have a constant ratio of target gene expression to reference gene expression. In these experiments, ß-actin is the most abundant protein in eukaryotic cells with constant expression [33]. The results of the calibrator-normalized quantification are expressed as the target/reference ratio of each sample divided by the target/reference ratio of the calibrator. The principle of this method is the determination of the relative amount of the target gene and the reference gene for each sample and for the calibrator. Quantification results are provided as normalized ratio ((target marker concentration [sample]/reference concentration [sample])/(target marker concentration [calibrator]/reference concentration [calibrator]). For each RNA marker investigated in this study, a standard curve was created, to be able to compare the unknown values of the patient samples to a standard value of a calibrator and to calculate the ratios relevant for quantification of the levels of mRNA expression. All experiments were performed in triple replications.

### 2.6. Statistical Analysis

Data are presented as arithmetic mean ± standard deviation (SD) for continuous variables or mean ± SEM for percent changes from baseline and as the number/proportion of patients for categorical variables. For the changes from baseline of the efficacy parameters, one-sided *P* values for within-group treatment differences were calculated, using the paired *t*-test procedure. In addition, ANOVA was performed with the baseline values of the observation parameters as covariates. Wilcoxon's two-sample test was used to calculate one-sided *P* values for between-group treatment differences. No transformations were applied to the secondary efficacy parameters. All inferential statistical analyses were performed in an exploratory sense, and all *P* values < 0.05 were interpreted as statistically significant. The authors had full access to the data and take responsibility for its integrity. All authors have read and agree to the paper as written.

## 3. Results

In total, 63 patients matching the inclusion and exclusion criteria could be included into this investigation (11 women, 52 men; age: 65.6±6.9 years (range: 45–77 years); disease duration: 6.6 ± 9.6 years (range: 0–58 years), HbA1c: 6.7±0.6%; BMI: 30.7 ± 4.2 kg/m²). All but one patient had a known prevalence of cardiovascular disease (98.4%), and 59 suffered from hypertension (93.6%). A total of 9 patients were current smokers (14.3%) and other 37 reported smoking in the past (58.7%). The study drugs were well tolerated, and all but one patient in the pioglitazone arm completed the study per protocol. This patient dropped out based on a personal decision after realizing a mismatch between personal schedules and study visit requirements. 

**Figure 1:**
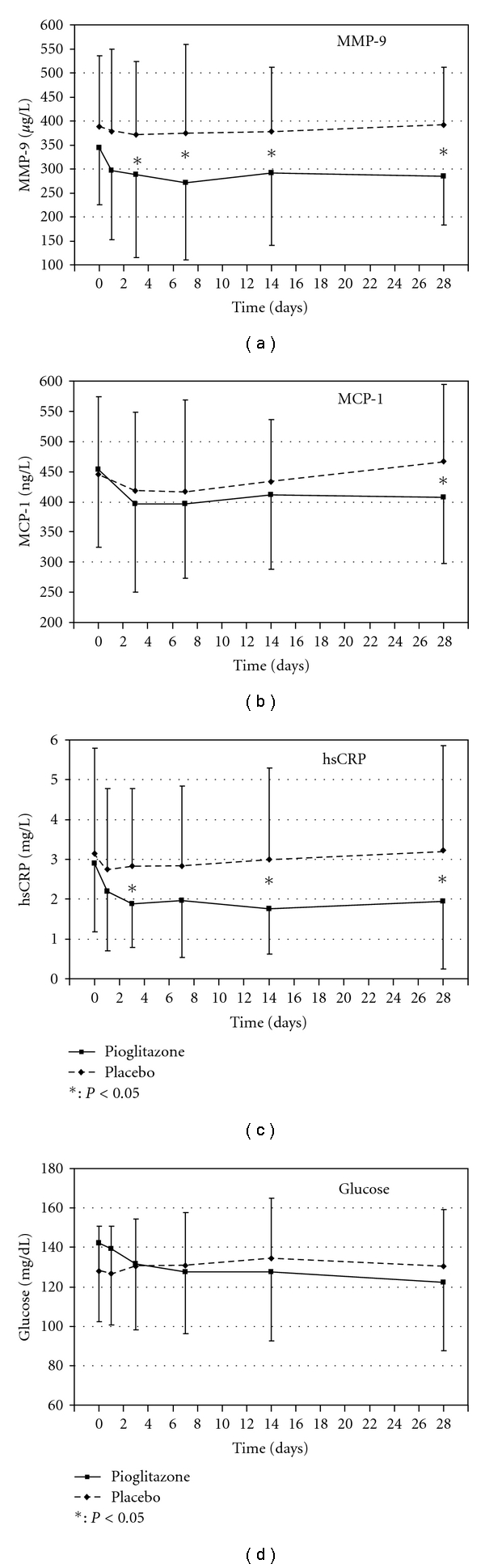
Changes in plasma concentrations of MMP-9, MCP-1, hsCRP, and glucose during the four-week observation period.

The change in fasting glucose concentrations and in the inflammatory cardiovascular risk markers MMP-9, MCP-1, and hsCRP during the observation period is provided in Figure 1. While a slight but nonsignificant decrease in fasting glucose could be observed with pioglitazone, the same group showed a fast and significant decrease in MMP-9 and hsCRP that was not seen in the placebo group. There was no significant change from baseline to endpoint in HbA1c in this well-controlled patient population in any of the two treatment groups, but a significant improvement in insulin resistance and the metabolic syndrome as indicated by a decrease in the HOMA_IR_ score, a decrease in intact proinsulin concentrations, and an increase in adiponectin values was observed in patients treated with pioglitazone (*P* < 0.001 versus placebo at endpoint in all cases). 

The mean absolute values for all determined plasma proteins and the other observation parameters at baseline and endpoint are provided in Table 2. There were significant improvements in many of these markers after 4 weeks of pioglitazone treatment, indicating an overall reduction of the inflammatory situation in the circulating blood, an improvement in endothelial and thrombocyte function, and an improvement in the metabolic risk situation. The differences from baseline to endpoint in the pioglitazone group and between the treatment groups at endpoint reached the level of statistical significance for many of the observation parameters. 

**Figure 2:**
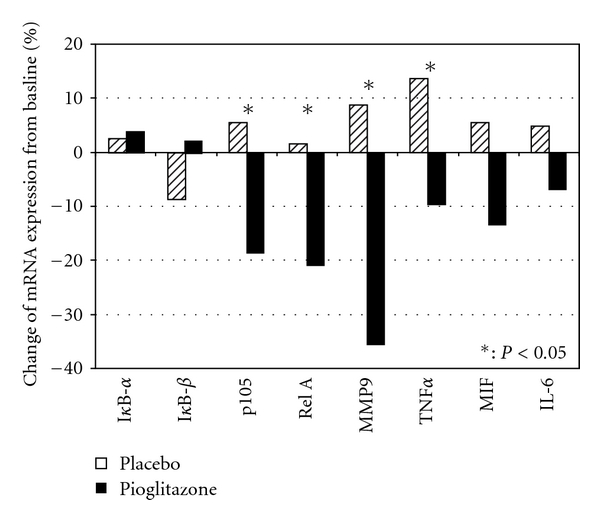
Percent changes in proinflammatory mRNA expression markers from baseline to week 4.

The quantification of the mRNA expression of the investigated proinflammatory cytokines in relation to a calibrator gene (ß-actin) is provided in Table 3. The relative expression of all proinflammatory markers increased in patients treated with placebo and decreased in patients on additional pioglitazone therapy, while the expression of the inhibitory markers changed inversely. The difference between the groups at endpoint was statistically significant for MMP9, TNF*α*, RelA, and p105. The percent changes in the mRNA expression of the observed biomarkers for both treatments are provided in Figure 2. The changes in MMP-9 mRNA expression were reflected by the corresponding protein concentrations. The overall expression pattern demonstrated a comprehensive decrease in the inflammatory state of the circulating monocytes during pioglitazone therapy, while a further increase of proinflammatory mRNA expression was observed with placebo. 

There were no clinically relevant adverse events observed in the study. In particular, there were no cases of severe hypoglycaemia reported. 

## 4. Discussion

The participants of our study had a good glycemic control by means of metformin and/or sulfonylurea drugs. The addition of pioglitazone induced a rapid reduction in the inflammatory expression state of circulating monocyte/macrophages, which went in parallel with a reduction of the plasma concentrations of corresponding plasma proteins and additional biomarkers for chronic inflammation in diabetic patients, which could not be observed in the placebo arm. Although the differences in mRNA marker expression between the two observation groups did not reach the level of statistical significance for all markers, the general expression pattern uniformly points into the direction of a comprehensive downregulation of macrophage activation by pioglitazone. The expression of the NF-*κ*B-related proteins (RelA and p105) and of NF-*κ*B-regulated proteins (TNF-*α*, MIF, and MMP-9) was significantly or slightly but nonsignificantly reduced, while the expression of the inhibitors to NF-*κ*B (*κ*B-*α* and I*κ*B-*β*) apparently increased. These anti-inflammatory effects preceded any possible effects on glycemia by pioglitazone, and the 4-week observation period was too short to observe a significant change in HbA1c in this trial. There were, however, several signs for an improvement in insulin resistance, ß-cell function, endothelial function, thrombocyte function, and the metabolic syndrome as indicated by appropriate changes in the corresponding laboratory biomarkers.

Our observations may provide an insight into the underlying cellular mechanisms of the short-term glucose-independent clinical effects of pioglitazone and rosiglitazone on endothelial and vascular function that were published recently in nondiabetic subjects and patients with type 2 diabetes. Hetzel and coworkers demonstrated that a three-week treatment with rosiglitazone did not change blood glucose or lipid levels of healthy subjects, but increased flow-mediated, endothelium-dependent vasodilatation starting already within the first day, which was paralleled by a rapid reduction of proinflammatory and prothrombotic biomarkers. They suggested a direct effect of PPAR*γ* activation on endothelial function and inflammation, independent of metabolic action [24].

Another group performed a randomized, placebo-controlled, double-blind crossover trial in 20 patients with type 2 diabetes on effective other oral anti-diabetic medication, to investigate the effect of treatment with 30 mg of pioglitazone on shear-stress-induced flow-mediated vasodilatation. After 4 weeks, they found an amelioration of endothelial function in conduit arteries irrespective of significant beneficial changes in the plasma levels of insulin, free fatty acids, adiponectin, or hsCRP [25]. Also, treatment with pioglitazone improved cutaneous microcirculation and endothelial function independent of glycemic control when compared with glimepiride [34]. 

While all these reports provide a conclusive clinical and biochemical picture regarding a fast anti-inflammatory and antithrombotic effect of PPAR*γ* activation, our results may contribute to a better understanding of these effects on a molecular basis. It is known that both abdominal fat and insulin resistance contribute to vascular disease, especially in obese patients. In particular, visceral fat contributes to inflammation and endothelial dysfunction through secretion of adipokines, like TNF*α* or IL-6, which are secreted by the lipid tissue after macrophage recruitment (through monocyte chemoattractant protein-1 (MCP-1) [11]. Pioglitazone has been demonstrated to decrease a variety of these adipokines in several clinical and experimental studies [19, 35, 36]. The decrease of several plasma proteins such as IL-6, TNF*α*, Angiotensin II, and the increase in adiponectin in our current experiment is also in line with these findings. However, our study does not clarify the mechanism by which pioglitazone may have down-regulated macrophage activation in such short time frame. Clinical studies addressing these effects have only been able to investigate the fat tissue as a whole, that is, including all different cellular subfractions. Fontana et al. were able to demonstrate by assessment of arteriovenous concentration differences with samples obtained from the portal vein that visceral fat is a clinically important site of IL-6 secretion, thus contributing to systemic inflammation [37]. It can be speculated from our results that at least part of the observed increase in proinflammatory cytokines may have derived from previously circulating mononuclear that had penetrated into the adipose tissue. 

One explanation for the observed results with pioglitazone could be a direct effect of the thiazolidinedione on mononuclear cells. It has been shown that PPAR*γ* has distinct functions in different cell types in the white adipose tissue, such that pioglitazone reduces macrophage infiltration by inducing apoptotic cell death specifically in macrophages through PPAR*γ* activation [38]. Since the macrophages recruited into the fat tissue are a major source of cytokines and proteins that are known to maintain systemic inflammation [11], a change in their inflammatory activity may be reflected by a downregulation of proinflammatory mRNA expression in the circulating mononuclear cells. 

Another contributor may also be an indirect effect of pioglitazone via modification of adipokine secretion derived from differentiating preadipocytes and other components of the lipid tissue. The complex nature of the interactions between lipid tissue, endothelium, muscle, liver, and the immune system in orchestrating systemic inflammation does not allow to draw ultimate conclusions from our observed effects of pioglitazone at this point, and further research is required to understand the underlying mechanisms. 

What might be clinical implications from our trial? The observed pleiotropic effects of pioglitazone occur fast and mainly independent of the metabolic effects of the drug. This finding may support an earlier and more frequent use of this drug in patients who are still well controlled with other “classical” anti-diabetic drugs but are at elevated risk for macrovascular disease. We have been able to demonstrate a significant decrease in surrogate markers for systemic inflammation and cardiovascular risk, including intima-media-thickness, insulin resistance, endothelial function, hsCRP, MMP-9, or MCP-1 with pioglitazone, while other anti-diabetic drugs resulting in an equal improvement of metabolic control had no such effects in diabetic patients [18, 19, 35]. Pioglitazone, when given by us in comparison or in addition to simvastatin, had an independent synergistic impact on the cardiovascular risk of patients with normoglycemic vascular insulin resistance [36, 39]. These clinical findings are in good agreement with our current observation of an overall down-regulation of the inflammatory state of circulating monocyte/macrophages by pioglitazone independent of glycemic control. 

In conclusion, the results of our study indicate that pioglitazone when given in addition to an effective antidiabetic treatment with metformin and/or sulfonylurea induced an overall decrease in plasma adipokines and in the inflammatory state of circulating mononuclear cells in patients with well-controlled type 2 diabetes mellitus, while a further deterioration was observed with placebo. These effects occurred independently from glycemic control and already after short treatment duration. Our findings may be helpful to understand the mechanism and nature of the multiple antiatherosclerotic and antithrombotic effects that have been reported in recent controlled clinical investigations and outcome trials comparing thiazolidinediones with other anti-diabetic drugs.

## Figures and Tables

**Table 1:** Primer composition and PCR protocols.

Gene	Forward primer Reverse primer	Annealing temperature [°C]	Annealing time [s]	Elongation time [s]	Product melting temperature [°C]
Rel-A	CAGTACCTGCCAGATACAGACGA GGGAAGGCACAGCAATG	63	10	7	88
P105	TGATGATTTACTAGCACAAGGAGACAT TGTACCCCCAGAGACCTCATAG	65	10	9	85
I*κ*B-*α*	CTGATGTCAATGCTCAGGAGCC TGTGTCATAGCTCTCCTCATCCTCAC	68	10	11	89
I*κ*B-ß	CTGAAAACTACGAGGGCCA CCTCCACTGCCAAATGAAG	64	5	8	91
TNF-*α*	CCCAGGGACCTCTCTCTAATC ATGGGCTACAGGCTTGTCACT	64	10	8	87
IL-6	CCCATGCAGGCACTTACTAC ACGTCTTCTTGAACCTCAGAACA	63	5	4	86
MIF	CGGACAGGGTCTACATCAA CTTAGGCGAAGGTGGAGTT	63	5	4	84
MMP-9	CCCATTTCGACGATGACGAGTTGTG GGAGTAGGATTGGCCTTGGAAGATG	64	10	13	92

**Table 2:** Clinical and biochemical observation parameters at baseline and after 4 weeks in both treatment arms.

	Pioglitazone		Placebo	
	Baseline	Endpoint	Baseline	Endpoint
HbA1c [%]	7.0 ± 1.1	6.8 ± 0.9	6.7 ± 0.6	6.6 ± 0.7
BMI [kg/m²]	31.0 ± 4.3	31.4 ± 4.5*	30.5 ± 4.1	30.4 ± 4.2
Systolic blood pressure [mmHg]	144 ± 15	137 ± 18*	141 ± 19	139 ± 20
Diastolic blood pressure [mmHg]	83 ± 11	80 ± 11	78 ± 11	79 ± 9
Waist/hip ratio	1.00 ± 0.05	1.00 ± 0.07	1.00 ± 0.06	1.00 ± 0.06
Glucose [mg/dL]	142 ± 40	122 ± 35***	128 ± 23	131 ± 29
Insulin [*μ*U/mL]	17 ± 10	12 ± 7***	18 ± 10	18 ± 11+
HOMA_IR_	5.9 ± 4.4	3.9 ± 2.6***	6.0 ± 3.5	6.4 ± 4.5+
Adiponectin [mg/dL]	8.7 ± 3.5	22.1 ± 9.1***	8.3 ± 4.7	8.2 ± 4.2^+++^
Intact proinsulin [pmol/L]	30 ± 37	19 ± 17*	24 ± 19	24 ± 20
hsCRP [mg/L]	2.9 ± 1.7	1.9 ± 1.7**	3.2 ± 2.6	3.2 ± 2.6^+^
MMP-9 [*μ*g/L]	344 ± 118	284 ± 101**	388 ± 147	391 ± 121^+++^
MCP-1 [*μ*g/L]	454 ± 130	406 ± 106*	446 ± 129	468 ± 127^+^
Total cholesterol [mmol/L]	4.63 ± 0.99	4.78 ± 1.09	4.60 ± 1.10	4.61 ± 1.00
LDL cholesterol [mmol/L]	2.46 ± 0.80	2.52 ± 0.76	2.21 ± 0.93	2.21 ± 0.90
HDL cholesterol [mmol/L]	1.18 ± 0.24	1.26 ± 0.26**	1.12 ± 0.21	1.13 ± 0.19^+^
Triglycerides [mmol/L]	1.84 ± 1.14	1.72 ± 0.99	2.41 ± 2.22	2.53 ± 1.73^+^
sICAM [*μ*g/L]	326 ± 90	319 ± 86	304 ± 56	310 ± 60
sVCAM [*μ*g/L]	915 ± 409	943 ± 452	797 ± 193	807 ± 220
sCD40L [*μ*g/L]	1.6 ± 1.8	1.1 ± 1.1	1.4 ± 1.2	1.0 ± 0.9
P-selectin [*μ*g/L]	95 ± 20	93 ± 22	96 ± 26	98 ± 26
IL-6 [ng/L]	3.3 ± 0.5	3.2 ± 0.1	3.3 ± 0.3	3.3 ± 0.4
Angiotensin II [*μ*g/L]	10.2 ± 9.2	8.0 ± 8.8*	9.2 ± 8.2	10.1 ± 10.1
Complement factor C3 [g/L]	1.6 ± 0.4	1.5 ± 0.3	1.6 ± 0.4	1.7 ± 0.5
MIF [*μ*g/L]	10.4 ± 5.3	9.6 ± 3.5	10.1 ± 4.1	10.3 ± 4.3

Within-group comparison: *: *P* < 0.05; **: *P* < 0.01; ***: *P* < 0.001 versus baseline.

Between groups for changes from baseline: ^+^: *P* < 0.05; ^++^: *P* < 0.01; ^+++^: *P* < 0.001.

**Table 3:** mRNA expression of NF*κ*B and NF*κ*B-modulated cytokines in circulating peripheral mononuclear cells at baseline and after 4 weeks of therapy with pioglitazone or placebo (reference gene: ß-actin).

	Pioglitazone		Placebo	
	Baseline	Endpoint	Baseline	Endpoint
p105 (p50 subunit of NF-*κ*B)	1.63 ± 0.80	1.33 ± 0.46*	1.35 ± 0.66	1.42 ± 0.91^+^
RelA (p65 subunit of NF-*κ*B)	1.20 + 0.74	0.95 ± 0.44*	1.05 ± 0.42	1.07 ± 0.45^+^
I*κ*B-*α*	1.28 ± 1.15	1.30 ± 1.14	1.13 ± 0.97	1.04 ± 0.75
I*κ*B-*β*	3.17 ± 2.05	3.30 ± 2.41	2.78 ± 1.63	2.86 ± 1.62
MMP-9	2.29 ± 2.68	1.48 ± 1.18	1.56 ± 2.02	1.69 ± 1.70^+^
TNF*α*	1.88 ± 1.20	1.70 ± 0.93	1.81 ± 0.94	2.02 ± 1.28^+^
MIF	0.98 ± 0.40	0.84 ± 0.31	0.83 ± 0.35	0.88 ± 0.52
IL-6	1.13 ± 0.52	1.05 ± 0.46	1.09 ± 0.57	1.14 ± 0.69

*: *P* < 0.05 (versus baseline); ^+^: *P* < 0.05 (between the groups for change from baseline).
